# Neuropsychiatric SLE in children with childhood-onset lupus nephritis: a 20-year retrospective cohort study

**DOI:** 10.1007/s00467-025-06904-0

**Published:** 2025-08-26

**Authors:** Matthew Lok-hei Wong, Ka-Man Yip, Alison Lap-tak Ma, Eugene Yu-hin Chan

**Affiliations:** 1 Department of Paediatric and Adolescent Medicine, Princess Margaret Hospital, Hong Kong, Hong Kong SAR; 2 Paediatric Nephrology Centre, Hong Kong Children’s Hospital, Hong Kong, Hong Kong SAR; 3 Department of Paediatrics and Adolescent Medicine, The University of Hong Kong, Hong Kong, Hong Kong SAR; 4 Department of Paediatrics, Faculty of Medicine, The Chinese University of Hong Kong, Shatin, Hong Kong SAR; 5 Hong Kong Hub of Pediatric Excellence (HOPE), The Chinese University of Hong Kong, Hong Kong, Hong Kong SAR

**Keywords:** Neuropsychiatric systemic lupus erythematosus, Lupus nephritis, Childhood onset systemic lupus erythematosus, CSLE, NPSLE

## Abstract

**Background:**

Neuropsychiatric systemic lupus erythematosus (NPSLE) and lupus nephritis (LN) are two major, life-threatening complications in childhood-onset SLE (cSLE). Data regarding the epidemiology and prognosis of children with concurrent NPSLE and LN remain scarce. This study aimed to investigate the clinical characteristics, associated factors, and outcomes of NPSLE in Chinese children with LN.

**Methods:**

A retrospective cohort study was conducted at the Paediatric Nephrology Centre of Hong Kong Children’s Hospital, including 95 Chinese children with biopsy-proven cLN. Comparisons were made between children with and without NPSLE.

**Results:**

Of 95 Chinese children with cLN, 11 (12%) developed NPSLE, and 31 NPSLE events were reported. Estimated glomerular filtration rate < 30 mL/min/1.73 m^2^ at diagnosis of LN (OR_adj_ 6.7, 95% CI 1.29–35.1) and higher maximal proteinuria during the observation period (OR_adj_ 1.07, 95% CI 1–1.13) were predictive of NPSLE upon multivariable analysis. Compared to children with LN who did not develop NPSLE, significantly more children who developed subsequent NPSLE flare following initial kidney involvement had a history of medication non-adherence (100% vs. 25%, *p* < 0.001), higher degree of proteinuria at the diagnosis of LN (urine protein/creatinine ratio, 5.7 vs. 2.4 mg/mg, *p* = 0.04) and during the entire observation period (urine protein/creatinine ratio, 13.2 vs. 3.3 mg/mg, *p* = 0.004). Patients with NPSLE had significantly lower complete remission rates for LN at 6- and 12-month post-induction (27.3% vs. 70.2%, *p* = 0.014; 45.5% vs. 83.3%, *p* = 0.01, respectively). Kaplan–Meier analysis showed that patients with NPSLE had worse kidney and patient survivals (log-rank test, *p* < 0.001, 0.0014, respectively) than those without NPSLE.

**Conclusions:**

Worse kidney and patient survivals are observed in cLN patients with NPSLE. Severe LN manifestation and medication non-adherence are associated with the development of NPSLE.

**Graphical Abstract:**

**Figure:**
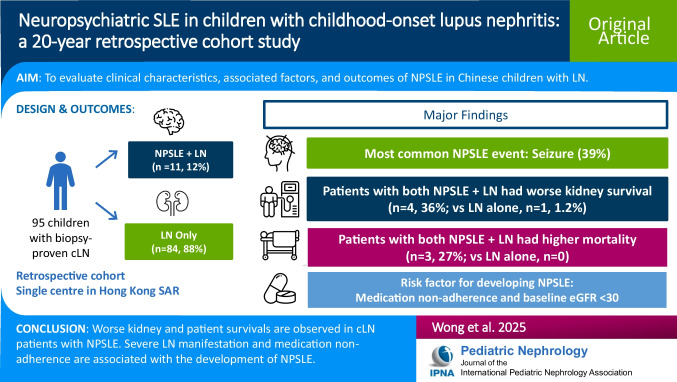
A higher resolution version of the Graphical abstract is available as Supplementary information

**Supplementary Information:**

The online version contains supplementary material available at 10.1007/s00467-025-06904-0.

## Introduction

Systemic lupus erythematosus (SLE) is a chronic autoimmune disease characterised by systemic inflammation affecting various organ systems. In childhood-onset SLE (cSLE), two of the most significant and life-threatening complications are neuropsychiatric systemic lupus erythematosus (NPSLE) and lupus nephritis (LN). NPSLE occurs in 25–50% of cSLE patients [1–3], with symptoms ranging from psychiatric to central and peripheral nervous system dysfunctions [1–5]. Kidney involvement, manifesting as LN, occurs in 35% of cSLE patients [6] and is associated with chronic kidney disease and kidney failure [7, 8]. Although the individual impacts of NPSLE and LN are well-recognised, there remains a significant knowledge gap regarding the epidemiology, clinical characteristics, and long-term prognosis of NPSLE in children with concurrent LN. Furthermore, the identification of associated factors for NPSLE can enable early patient stratification to inform individualised treatments [9]. Consequently, this study aimed to evaluate the characteristics, associated factors, and outcomes of NPSLE in Chinese children with LN.

## Methods

### Study design and patient selection

We conducted a single-centre retrospective cohort study at the Paediatric Nephrology Centre of Hong Kong Children’s Hospital, the designated referral centre for complicated kidney disease for all children in Hong Kong. We included all Chinese children with biopsy-proven LN diagnosed before 18 years of age, between 1 June 2000 to 31 May 2021. Exclusion criteria included lack of histological LN confirmation, LN diagnosis after 18 years of age, or alternate explanations for neuropsychiatric symptoms. The diagnosis of cSLE was based on 1997 American College of Rheumatology revised criteria for the classification of systemic lupus erythematosus or 2012 Systemic Lupus International Collaborating Clinics criteria [10, 11]. Kidney histology was classified using the 1982 World Health Organization classification (before 2004) or the International Society of Nephrology/Renal Pathology Society classification (after 2004) [12, 13]. NPSLE was diagnosed by paediatric nephrologists in collaboration with paediatric rheumatologists, neurologists, psychiatrists, and clinical psychologists using the American College of Rheumatology nomenclature for NPSLE [14]. Patients presenting with isolated headaches, anxiety, or mood disorder were excluded due to their non-specific nature [2]. Concurrent NPSLE event was defined as symptom onset within 1 month before or after the diagnosis of LN. This study was approved by the Central Institution Review Board of the Hospital Authority, Hong Kong (Ref. PAED-2022–009).

### Clinical evaluation

Data on patient demographics, clinical presentations, investigations, treatment, and outcomes were retrospectively obtained from electronic medical records. Patients were followed every 1–8 weeks with clinical symptoms and physical findings, and investigation results were documented [15]. Neurological investigations for suspected NPSLE were performed at physicians’ discretion.

### Treatment

Treatment of LN included induction and maintenance of immunosuppressive therapy. For induction, proliferative LN was treated with oral prednisolone (0.8–1 mg/kg/day) with or without intravenous pulse methylprednisolone (10–30 mg/kg/dose for three doses). In addition, patients received mycophenolate mofetil (MMF) (1200 mg/m^2^/day), or for severe cases, they received intravenous cyclophosphamide (CYC) according to either the National Institutes of Health (NIH) protocol (0.5–1 g/m^2^/month for 6 months) or the Euro-lupus protocol (500 mg every 2 weeks for six infusions).

The decision to use CYC was based on clinical presentations, typically for patients with organ-threatening presentation with rapidly deteriorating kidney function, histological evidence of severe inflammatory activity (e.g. extensive crescents), or severe concurrent extra-renal manifestations like NPSLE with cerebral vasculitis. A small number of patients received oral CYC for 6 months or azathioprine as induction therapy. For maintenance therapy, patients received a tapering course of oral prednisolone with MMF or azathioprine. Hydroxychloroquine and renin–angiotensin–aldosterone-system inhibitors were prescribed unless contraindicated. Membranous LN was treated with corticosteroid and MMF. Adjunctive therapy, e.g. rituximab, intravenous immunoglobulin, and plasma exchange or triple therapy (corticosteroid, MMF, and calcineurin inhibitors), was used in selected patients.

In most patients with NPSLE, induction therapy included intravenous CYC (NIH regimen) and corticosteroids, followed by corticosteroids with MMF or azathioprine as maintenance. Adjunctive therapy including rituximab [16] and intravenous immunoglobulin was used in selected cases. Patients suffering from ischaemic stroke or anti-phospholipid syndrome received additional anti-platelets or anti-coagulants, whereas those with seizures were prescribed anti-seizure medications. The assessment of medication adherence was conducted at every clinic visit. Poor adherence to medication was self-reported and defined as taking < 80% of the prescribed medications between consecutive follow-up [17].

### Outcomes

Primary outcome included the epidemiology and clinical characteristics of NPSLE in the background of cLN. Secondary outcomes included associated risk factors and long-term outcomes of NPSLE. Long-term outcomes included the treatment response and relapse of disease, patient, and kidney survival. Response to treatment for LN was recorded at 6 and 12 months following initial therapy. Complete response (CR) was defined as persistent urine protein-creatinine ratio (UPCR) < 0.5 mg/mg in early morning urine, while partial response (PR) was defined as UPCR decreases by ≥ 50% and to < 3 mg/mg in early morning urine [18]. Nonresponse was defined as patients who failed to attain complete or partial response [18]. Kidney relapse was defined as UPCR > 1 mg/mg in patients with baseline UPCR < 0.5 mg/mg or rise in UCR by ≥ 1 mg/mg in those with baseline UPCR > 0.5 mg/mg, or an increase in serum creatinine by 15% or more compared from baseline without alternative explanation, and with supporting evidence such as increased serological activity or activity on kidney histology [19]. Kidney survival was defined as the survival of patients without the need for initiating kidney replacement therapy.

### Statistical analysis

Statistical analysis was performed by IBM SPSS statistics version 27 software. Patient characteristics were examined by descriptive statistics. Comparison between patients with and without NPSLE in demographics, investigations, and treatment was conducted using Fisher’s exact test or Pearson’s chi-squared test for categorical variables, while Student’s *t*-test or Mann–Whitney *U*-test was used for the comparison of continuous data. Univariable logistic regression models were built to identify factors associated with developing NPSLE, followed by multivariable regression analysis with the significant variables identified, to investigate their combined effects on the outcome. To analyse the survival curve and survival pattern for patient and kidney survival, Kaplan–Meier survival curves were used. Log-rank test was used to examine the significant difference in survival rates between LN patients with and without NPSLE. All tests were two-tailed and conducted with 5% alpha levels.

## Results

### Patient characteristics

**Fig. 1:**
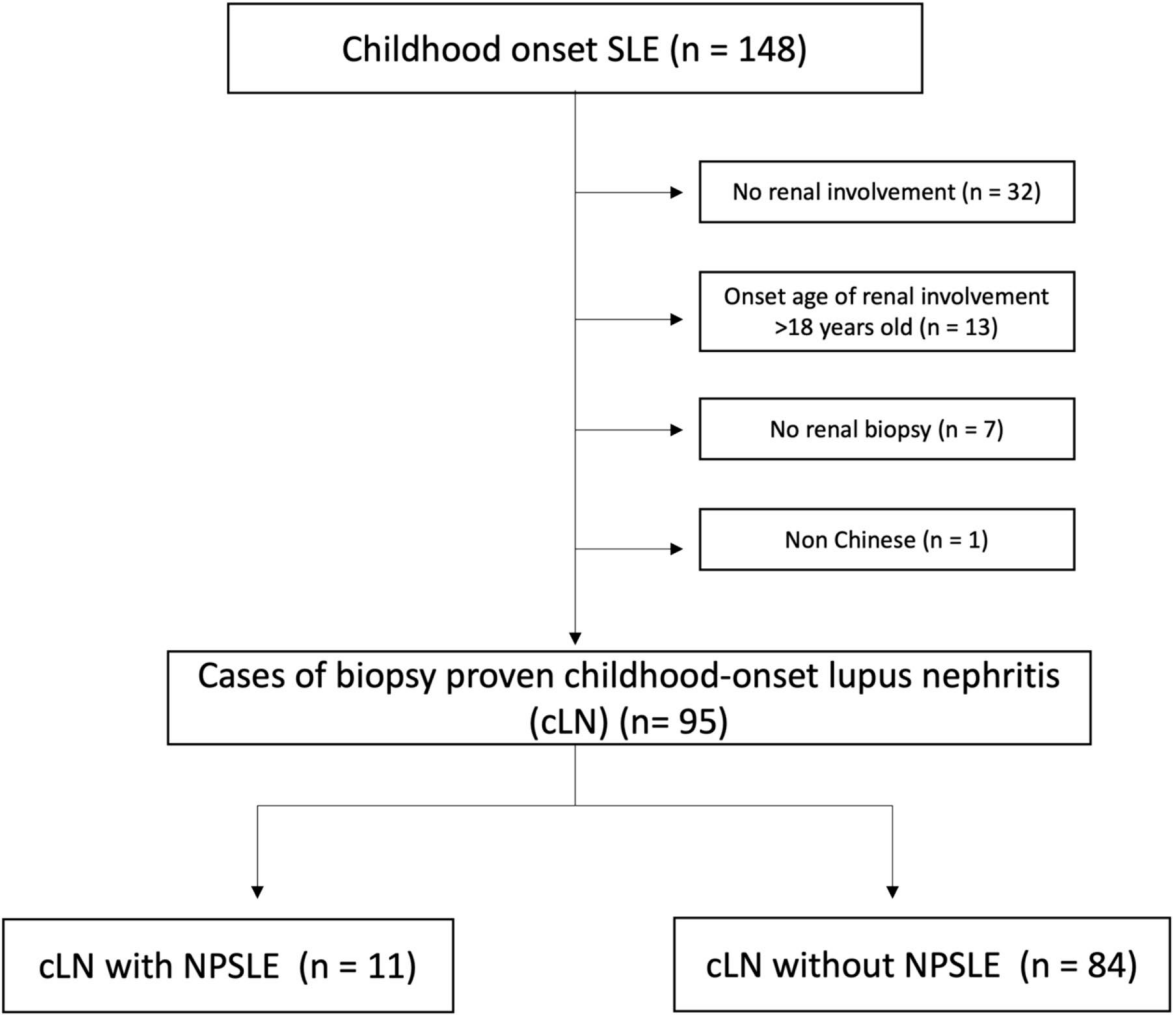
Patient flow chart

A total of 148 cSLE patients were identified. Of these patients, 95 Chinese children with biopsy-proven cLN (Fig. 1) (82% female; mean age at LN diagnosis 13.3 ± 3.2 years) were included. Mean observation period was 7.8 ± 3.9 years. A total of 84 patients (88%) had proliferative LN. Overall, 11 children (12%) developed NPSLE (64% females; mean age at NPSLE presentation 14.7 ± 4.2 years). Six (55%) children developed NPSLE after the diagnosis of LN, and one (9%) and four (36%) patients developed NPSLE before and at the time of LN presentation, respectively. Baseline demographics and clinical characteristics are presented in Table 1 and Supplementary Table S1.

**Table 1:** Baseline characteristics of 95 children with biopsy-proven lupus nephritis

	Total (*N* = 95)	With NPSLE (*N* = 11)	Without NPSLE (*N* = 84)	*P* value
Sex				
Female	78 (82%)	7 (64%)	71 (85%)	NS
Male	17 (18%)	4 (36%)	13 (15%)	
Age of cSLE diagnosis (years)	13.2 +/− 3.2	13.3 +/− 3.9	13.2 +/− 3.2	NS
Age of LN diagnosis (years)	13.3 +/− 3.2	13.3 +/− 3.9	13.3 +/− 3.1	NS
Age of first NPSLE manifestation (years)		14.7 ± 4.2	/	
Observation period (years)	7.8 +/− 3.9	6.6 +/− 4.3	7.9 +/− 3.9	NS
Histological classifications				
* II *	1 (1%)	0	1 (1%)	NS
*III*	21 (22%)	1 (9%)	20 (24%)	
*IV*	45 (47%)	7 (64%)	38 (45%)	
*V *	9 (9.5%)	0	9 (11%)	
*V + III/IV*	18 (19%)	2 (18%)	16 (19%)	
*Podocytopathy*	1 (1%)	1 (9%)	0	
Activity index score^a^	8 (4–10)	6.5 (4–9)	8 (4–10)	NS
Chronicity index score^a^	0 (0–2)	0 (0–2)	0.5 (0–2)	NS
Kidney involvement				NS
AKI with dialysis	7 (7%)	2 (18%)	5 (6%)	
Nephritic/nephrotic syndrome	66 (70%)	9 (82%)	57 (68%)	
Proteinuria	22 (23%)	0	22 (26%)	
Onset of first NPSLE manifestation				
Before LN	1 (1.1%)	1 (9%)	/	
Concurrent with LN	4 (4.2%)	4 (36%)	/	
After LN	6 (6.3%)	(55%)	/	
Time after LN onset (years)	2.6 (0.2–5.3)	2.6 (0.2–5.3)	/	
Induction therapy				NS
Methylprednisolone	62 (65%)	9 (82%)	53 (63%)	
Prednisolone	33 (35%)	2 (18%)	31 (37%)	
Initial-maintenance therapy				NS
Aza-Aza	14 (15%)	0	14 (17%)	
Aza-MMF	1 (1.1%)	0	1 (1.2%)	
CYC-Aza	17 (18%)	2 (18%)	15 (18%)	
CYC-MMF	16 (17%)	2 (18%)	14 (17%)	
MMF-MMF	39 (41%)	6 (55%)	33 (39%)	
Triple therapy^**c**^	5 (5.3%)	0	5 (6%)	
CNI-CNI	1 (1.1%)	0	1(1.2%)	
Other	2 (2.1%)	1 (9.1%)	1 (1.2%)	
Other maintenance therapy				
Anti-malarials	78 (82%)	8 (73%)	70 (83%)	NS
Adjunctive therapy				
IVIg	3 (3.2%)	1 (9.1%)	2 (2.4%)	NS
Rituximab	8 (8.4%)	1 (9.1%)	7 (8.3%)	NS
Plasmapharesis	2 (2.1%)	0	2 (2.4%)	NS
Medical nonadherence	27(28%)	6 (55%)	21 (25%)	NS

Value expressed as count (%), mean (+/− SD), median (IQR) ^a^Had missing data ^c^Triple therapy included: CNIs in addition to corticosteroid and MMF*NS*, not statistically significant, *p* value > 0.05; *cSLE*, childhood onset systemic lupus erythematosus; *NPSLE*, neuropsychiatric systemic lupus erythematosus; *GI*, gastrointestinal; *AKI*, acute kidney injury; *APS*, anti-phospholipid syndrome; *CYC*, cyclophosphamide; *Aza*, azathioprine; *MMF*, mycophenolate mofetil; *CNI*, calcineurin inhibitor; *IVIg*, intravenous immunoglobulin

### NPSLE patient characteristics

Of the 11 patients, 10 (91%) experienced more than one NPSLE event. A total of 31 NPSLE events were recorded. The most common neuropsychiatric manifestation was seizure (*n* = 12, 39%), followed by acute confusional state (*n* = 6, 19%) and cerebrovascular accidents (*n* = 5, 16%) (Table 2).

**Table 2:** Neuropsychiatric manifestations in 11 of 95 lupus nephritis patients

	NPSLE patients (***N*** = 11)	Events^c^ (***N*** = 31)
*Central nervous system*		
Aseptic meningitis	0	0
Cerebrovascular disease	5 (46%)	5 (16%)
*Ischemic stroke*	3 (27%)	3 (10%)
*Intracranial haemorrhage*	2 (18%)	2 (6.5%)
Demyelinating syndrome	0	0
Headache^a^	1 (9.1%)	1 (3.2%)
Movement disorder (chorea)	0	0
Myelopathy	1 (9.1%)	1 (3.2%)
Seizure disorder^b^	6 (55%)	12(39%)
*Generalised*	4 (36%)	7 (23%)
*Partial*	3 (27%)	5 (16%)
Acute confusional state	6 (55%)	6 (19%)
Anxiety disorder^a^	1 (9%)	1 (3%)
Cognitive dysfunction	2 (18%)	2 (6%)
Mood disorder^a^	1 (9%)	1 (3%)
Psychosis	0	0
Guillain-Barré syndrome	0	0
Autonomic disorder	0	0
Mononeuropathy (single/multiplex)	0	0
Myasthenia gravis	0	0
Cranial neuropathy	2 (18%)	2 (6%)
Plexopathy	0	0
Polyneuropathy	0	0

Value expressed as count (%) ^a^Isolated headache, anxiety and mood disorder without other NPSLE manifestations were excluded ^b^One patient had both generalised and partial seizure ^c^One patient can have more than 1 NPSLE events

*NPSLE*, neuropsychiatric systemic lupus erythematosus

All 11 NPSLE patients had abnormalities identified on CT or MRI brain, with cerebral infarct (*n* = 4, 36%) being the most common finding. CSF protein was elevated in 60% (*n* = 3/5) of patients, while white blood count was normal in all CSF samples. Nerve conduction study was performed in one patient which was unremarkable. Electroencephalogram showed slow wave in all four patients with encephalopathy or seizure (Supplementary Table S2).

### Summary of NPSLE treatments

Of the 11 patients with NPSLE, pulse intravenous methylprednisolone and intravenous cyclophosphamide were used in 10 patients, respectively. Two patients (27%) received add-on rituximab due to treatment-refractory disease, whereas another two patients received intravenous immunoglobulin for severe thrombocytopenia. For maintenance therapy, all patients were treated with oral prednisolone, whereas eight patients (73%) received MMF and two patients (18%) received azathioprine. One patient passed away before the commencement of maintenance immunosuppressive therapy. Four patients (36%) suffering from ischemic stroke or antiphospholipid syndrome received antiplatelet agents or anticoagulants, whereas six patients with seizure were prescribed with anti-epileptics. Table 3 summarises NP manifestations, treatment, and outcome of each NPSLE patient.

**Table 3:** Manifestations, treatment, and outcome of each NPSLE patient

Number	Sex	Age at LN diagnosis (years)	Age at first NPSLE manifestations (years)	First NPSLE event occurred before/concurrent/after onset of LN	NPSLE manifestations	Neurological investigation	Treatment	Mortality	Neurological outcome	Kidney outcome
1	F	15.5	16.8	After	Seizure and delirium	CT brain: vasculitis, MRI brain: normal	Induction: IV MP, IV CYC; rituximab; maintenance: prednisolone, MMF; anti-convulsants, anti-platelet agent	No	Complete neurological recovery	Kidney failure, received kidney transplant, graft failure requiring HD
2	F	17.6	11.3	Before	ICH: left hemiplegia and GTC	CT brain: SAH, ICH, hydrocephalus, mild mid line shift; MRI brain: ICH	Craniotomy; induction: IV MP, IVIG; maintenance: prednisolone, Aza; anti-convulsants	No	Complete neurological recovery	Kidney failure, received PD
3	F	8.8	8.8	Concurrent	GTC, delirium and severe headache, cognitive dysfunction	MRI brain: vasculitic changes	Induction: IV MP, IV CYC; maintenance: prednisolone, MMF; anti-convulsants	Died 13.6 years after diagnosis of LN	Death due to severe culture negative pneumonia	CKD stage 4
4	M	11.7	17	After	Ischemic stroke: hemiplegia, limbs numbness and quadrantanopia	CT brain: multiple infarction; MRI brain: multiple infarctions, vasculitis	Induction: oral prednisolone, IV CYC; maintenance: MMF; anti-coagulant	No	Complete neurological recovery	CKD stage 2
5	F	8.7	19.5	After	Acute delirium	CT brain: vasculitic changes; MRI brain: vasculitic change	Induction: IV MP, IV CYC; maintenance: prednisolone, MMF	No	Complete neurological recovery	CKD stage 2
6	F	16	19.9	After	Bilateral vertical gaze palsy with painful intraoral numbness, unilateral sensorineural hearing loss	CT brain: normal; MRI brain: inflammatory changes over midbrain	Induction: IV MP, IV CYC; maintenance: prednisolone, MMF; anti-platelet agent	No	Residual unilateral sensorineural hearing loss	CKD stage 2
7	F	15.6	15.8	After	Recurrent GTC	CT brain: cortical infarction MRI brain: normal; EEG: slow wave	Induction: IV MP, IV CYC; maintenance: prednisolone, MMF; anticonvulsants	No	Complete neurological recovery	No CKD
8	F	6.6	6.8	After	Focal seizure, loss of consciousness, delirium, dystonia	CT brain: cerebral atrophy; MRI brain: cerebral atrophy, ischemic changes; MRI spine: chronic atrophy changes; EEG: slow wave	Induction: IV MP, IV CYC; rituximab, IVIG; maintenance: prednisolone, MMF; anticonvulsants	No	Persistent dystonia with limb contractures	No CKD
9	M	17.4	17.4	Concurrent	ICH: mood changes, acute delirium and loss of consciousness	CT brain: vasculitic changes; MRI brain: ICH, transtentorial and cerebellar herniation; EEG: slow wave	Craniectomy; induction: IV MP, IV CYC; IVIG, anakinra; maintenance: prednisolone^a^	Died at first month of diagnosis	Death due to NPSLE and MAS	CKD stage 3 before death
10	M	15	15	Concurrent	Ischemic stroke: unilateral facial, trunk and limb numbness with quadrantanopia. Anxiety and worsening memory	CT Brain: lacunar infarct; MRI brain: vasculitic changes, infarction, dural sinus thrombosis	Induction: IV MP, IV CYC; rituximab; maintenance: prednisolone, MMF; anticoagulant	No	Some residual cognitive dysfunction	CKD stage 2
11	M	13	13	Concurrent	Ischemic stroke: delirium, hemiplegia, ataxia, ptosis, recurrent focal seizure. LMN facial palsy	CT brain: infarction; EEG: slow wave	Induction: IV MP, IV CYC; maintenance: prednisolone, MMF; anticonvulsants	Died at first month of diagnosis	Death due to NPSLE	CKD stage 3 before death

*LN*, lupus nephritis; *NPSLE*, neuropsychiatric systemic lupus erythematosus; *CT*, computed tomography; *MRI*, magnetic resonance imaging; *MP*, methylprednisolone; *CYC*, cyclophosphamide; *GTC*, generalised tonic clonic seizure; *ICH*, intracranial haemorrhage; *SAH*, subarachonoid haemorrhage; *IVIG*, intravenous immunoglobulin; *Aza*, azathioprine; *PD*, peritoneal dialysis; *CKD*, chronic kidney disease; *MMF*, mycophenolate mofetil; *EEG*, electroencephalography; *LMN*, lower motor neuron type

### Risk factors for developing NPSLE

Comparing children with LN only, more children with both LN and NPSLE presented with worse kidney function with eGFR < 30 mL/min/1.73 m^2^ at the diagnosis of LN (27% vs. 6%, *p* = 0.048) and higher maximal throughout the observation period (UPCR, 6.6 vs. 3.3 mg/mg, *p* = 0.044) (Supplementary Table S3). There was no significant difference between the two groups regarding baseline demographics, haematological parameters, complement profiles, antibodies profiles, and therapy regimen (Table 1; Supplementary Tables S1, S3). Multivariable analysis by logistic regression showed that eGFR < 30 mL/min/1.73 m^2^ at LN diagnosis (OR_adj_ 6.7, 95% CI 1.29–35.1, *p* = 0.02) and higher maximal proteinuria recorded during the observation period (OR_adj_ 1.06**,** 95% CI 1–1.13, *p* = 0.04) were significant predictive factors for the development of NPSLE (Table 4).

**Table 4:** Logistic regression analysis on risk factors of developing neuropsychiatric manifestations in children with lupus nephritis

Univariable analysis on risk factors of developing NPSLE in LN children		
	Odd ratio (95% CI)	*P* value
*Investigations at diagnosis of LN*		
Urine protein creatinine ratio (mg/mg)	1.23 (1.03–1.46)	NS
Serum creatinine (umol/L)	1 (0.99–1.01)	NS
eGFR < 30 (mL/min/1.73 m^2^)	**5.85 (1.17–29.1)**	*0.03
Anti-dsDNA (IU/mL)	0.997 (0.99–1.00)	NS
C3 (g/L)	0.89 (0.22–3.61)	NS
ESR (mm/h)	0.983 (0.96–1.01)	NS
*Laboratory parameters during follow up*		
Highest urine protein creatinine ratio (mg/mg)	**1.06 (1–1.13)**	*0.05
Highest serum creatinine (umol/L)	1 (0.996–1.01)	NS
Highest anti-dsDNA (IU/mL)	0.998 (0.99–1.004)	NS
Lowest C3 (g/L)	0.32 (0.01–12.6)	NS
Lowest platelet × 10^9^/L	0.99 (0.98–1.00)	NS
Multivariable analysis on risk factors of developing NPSLE in LN children		
	Adjusted odd ratio (95% CI)	*P* value
eGFR < 30 mL/min/1.73 m^2^at diagnosis of LN	**6.7 (1.29–35.1)**	*0.02
Highest urine protein creatinine ratio (mg/mg)	**1.07 (1.00–1.13)**	*0.04

Value expressed as count (%), median (IQR) ^*^p < 0.05: statistically significant; *NS*, not statistically significant, *p* value > 0.05

*NPSLE*, neuropsychiatric systemic lupus erythematosus; *LN*, lupus nephritis; *eGFR*, estimated glomerular filtration rate; *LA*, lupus anticoagulant; *ESR*, erythrocyte sedimentation rate; *anti-dsDNA*, anti-double stranded DNA titre

We further compared the six patients who developed disease flare of NPSLE after initial LN presentation against those who did not. In addition to a higher degree of maximal proteinuria during the observation period, patients who subsequently developed NPSLE had a higher rate of medication non-adherence (100% vs. 25%, *p* < 0.001) (Table 5).

**Table 5:** Subgroup analysis in children with neuropsychiatric manifestations after diagnosis of LN

	NPSLE after diagnosis of LN (*N* = 6)	Without NPSLE (*N* = 84)	*P* value
*Laboratory parameters at diagnosis of LN*			
Urine protein creatinine ratio (mg/mg)	5.7 (2.8–11.9)	2.4 (0.8–5.5)	*0.039
Serum creatinine (umol/L)	59 (50.5–115)	56 (44–81)	NS
eGFR < 30 (mL/min/1.73 m^2^)	2 (33%)	5 (6%)	NS
*Laboratory parameters during follow-up*			
Highest urine protein creatinine ratio (mg/mg)	13.2 (4.8–36.1)	3.3 (1.7–6.7)	*0.004
Highest serum creatinine (umol/L)	90 (77–133)	83 (64–128)	NS
Medication non-adherence	6 (100%)	21 (25%)	* < 0.001

Value expressed as count (%), median (IQR)^*^p < 0.05: statistically significant

*NS*, not statistically significant, *p*value > 0.05; *NPSLE*, neuropsychiatric systemic lupus erythematosus; *LN*, lupus nephritis; *eGFR*, estimated glomerular filtration rate

### Neurological, kidney, and patient outcomes

Of eleven patients with NPSLE, five patients (45%) had complete neurological recovery. Three (27%) patients had neurological sequelae, including one patient who had persistent dystonia with limb contracture, one patient who had residual unilateral sensorineural hearing loss, and one patient who had residual cognitive dysfunction. Three patients (27%) died.

**Fig. 2:**
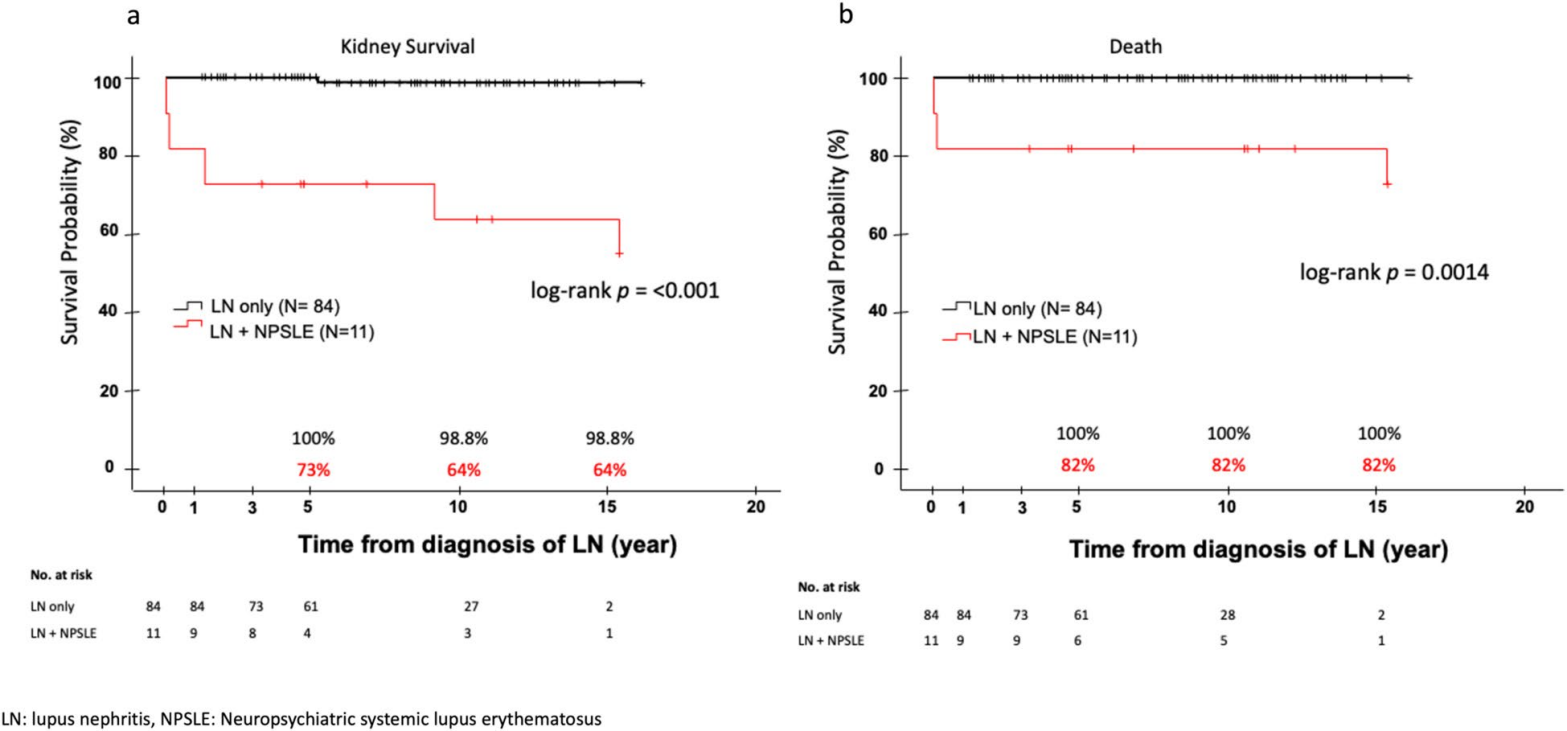
**a** Kaplan–Meier curve on kidney survival in LN children with and without NPSLE **b** Kaplan–Meier curve on survival in LN children with and without NPSLE

Patients with NPSLE had significantly lower complete response rate for LN at 6 (27% vs. 70%, *p* = 0.014) and 12 months (46% vs. 83%, *p* = 0.01) post-induction compared to those without NPSLE (Table 6). A significantly higher proportion of patients with NPSLE developed CKD (64% vs. 26%, *p* = 0.03) and kidney failure (18% vs. 1.2%, *p* = 0.035) compared to those without NPSLE. There were three deaths (3.2%) recorded in the cohort and all of them had NPSLE. The mortality rate was higher in children with NPSLE (27% vs. 0%, *p* = 0.001) (Table 6). Worse kidney survival was observed among patients with NPSLE (1 year, 82% vs. 100%; 3 years 73% vs. 100%, 5 years, 73% vs. 100%; 10 years, 64% vs. 99%; 15 years, 64% vs. 99%; *p* < 0.001) (Fig. 2a). Children with NPSLE also had an inferior patient survival rate than patients without NPSLE (1 year 82% vs. 100%; 3 years 82% vs. 100%; 5 years, 82% vs. 100%; 10 years, 82% vs. 100%; 15 years, 82% vs. 100%; *p* = 0.0014) (Fig. 2b). There were no significant differences between the two groups for serum creatinine, UPCR, and eGFR at last follow-up (Table 6).

**Table 6:** Comparison of outcome in lupus nephritis children with and without neuropsychiatric manifestations

Total (*N* = 95)	With NPSLE (*N* = 11)	Without NPSLE (*N* = 84)	*P* value	
Remission at 6 months				
*Complete response*	62 (65%)	3 (27%)	59 (70%)	*0.014
*Partial response and non-response*	33 (35%)	8 (73%)	25 (30%)	
Remission at 12 months				
*Complete response*	75 (79%)	5 (46%)	70 (83%)	*0.01
*Partial response and non-response*	20 (21%)	6 (55%)	14 (17%)	
Kidney relapse	37 (39%)	6 (55%)	31 (37%)	NS
Development of CKD				
*No CKD*	66 (70%)	4 (36%)	62 (74%)	*0.031
*CKD stages 2–5*	29 (31%)	7 (64%)	22 (26%)	
Kidney failure	3 (3.2%)	2 (18%)	1 (1.2%)	*0.035
Death	3 (3.2%)	3 (27%)	0	*0.001
Kidney parameters at last follow-up				
*Serum creatinine (umol/L)*	56 (48–69)	74 (50–112)	56 (48–67)	NS
*eGFR* (mL/min/1.73 m^2^)	103 (84–115)	79 (55–112)	104 (87–115)	NS
*Urine protein creatinine ratio (mg/mg)*	0.1 (0.1–0.5)	0.3 (0.1–0.8)	0.1 (0.1–0.4)	NS

Value expressed as count (%), median (IQR). *p < 0.05: statistically significant

*NS*, not statistically significant, *p* value > 0.05; *NPSLE*, neuropsychiatric systemic lupus erythematosus; *eGFR*, estimated glomerular filtration rate; *CKD*, chronic kidney disease

## Discussion

We found that 12% of children with cLN developed NPSLE, which was associated with severe kidney manifestations and medication non-adherence. Importantly, the presence of neurological manifestation in cLN correlated with worse kidney and patient survival, highlighting the need for timely immunosuppressive therapy to optimise patient outcomes.

Overall, 12% of our patient cohort suffered from both LN and NPSLE. Our incidence appeared to be lower than those reported in other paediatric populations with cLN (14.5 to 37%) [9, 20]. Such a discrepancy could be attributed to patients’ different ethnicity and heterogeneous definitions for NPSLE. We adopted a strict definition of NPSLE, excluding patients with isolated headache, anxiety, or mood disorder [2]. Importantly, all these NPSLE events were symptomatic and confirmed on neuroimaging. These events were on the more severe spectrum of NPSLE that warrant urgent attention and aggressive immunosuppressive therapy.

The presentation of NPSLE could be variable, and one of our patients presented with bilateral vertical gaze palsy with complete recovery after immunosuppressive therapy with intravenous pulse methylprednisolone and cyclophosphamide [21]. Only 5 out of 11 patients with NPSLE (45%) were able to attain complete neurological recovery in our cohort. This figure was lower than those reported in other cohort studies (84 to 88%) [2, 3]. The neurological outcome of children with both LN and NPSLE was not well-reported previously. The lower rate of complete neurological recovery observed in our study could be attributed to more severe NPSLE as well as the presence of kidney involvement in all our cases.

Comparing children with LN only, patients with both LN and NPSLE had more severe kidney manifestations, including low eGFR < 30 mL/min/1.73 m^2^ at diagnosis of LN and higher degree of proteinuria during the follow-up period. To our knowledge, these findings were not well-reported previously. Proteinuria was well known to be an important prognostication marker for LN [22]. On the other hand, lower baseline eGFR at presentation of LN was associated with poorer kidney outcome [23]. This observation likely implies that children with more severe kidney manifestations would have higher overall disease activity, hence an elevated risk of developing extra-renal manifestations including NPSLE.

Another important observation is that medication non-adherence was common in NPSLE patients, with 55% of them not adhering to their prescribed medications. This highlights the importance of improving drug compliance as a modifiable risk factor for developing NPSLE, especially among young people, where the rates of non-adherence could be up to 65% [24]. Non-adherence has been reported to be associated with increase in disease flares and hospitalisations [25, 26], while NPSLE symptoms like depression and anxiety disorder would worsen the adherence.

We demonstrated that patient and kidney survival were worse in cLN patients with NPSLE. All three mortalities in the cohort suffered from both LN and NPSLE. The mortality rate of 27% in our cohort was higher than other NPSLE cohorts [2, 27] due to potential referral bias as we are the territory-wide referral for complicated paediatric kidney conditions and all our patients had LN which is considered to be a severe manifestation of cSLE. Vyas et al. similarly reported a higher mortality rate among African American children who developed both LN and NPSLE (35%) compared to those with LN only [9]. Moreover, a recent large multi-centre cSLE cohort study in Brazil showed that both NPSLE and CKD increased risk of mortality [28].

Another important finding in our study was the association between NPSLE and worse kidney outcome with significantly higher rates of CKD (64% vs. 26%) and kidney failure (18% vs. 1.2%). Vyas et al. also found that children with both NPSLE and LN were five times more likely to progress to kidney failure than those with LN only [9]. A large cLN cohort study in China also demonstrated that NPSLE was an independent risk factor for poor kidney prognosis [20]. Notably, our study found that LN children with NPSLE were associated with lower complete remission rate at 6 and 12 months after treatment for LN, compared with LN patients without NPSLE (27.3% vs. 70.2%, 45.5% vs. 83.3%, respectively). These suggest the presence of NPSLE in cLN indicates high disease activity and may be more refractory to standard immunosuppressive therapy. Add-on therapy, such as rituximab, may be considered to optimise outcomes in this specific patient population [16].

There are a few limitations in our study. First, our study was limited by a small sample size, and only a few patients developed NPSLE (*n* = 11), resulting in unequal group sizes. This limitation reduces the statistical power and generalisability of our findings. Therefore, the relevant analyses should be considered exploratory in nature. Furthermore, the reported rate of NPSLE could be underestimated because not all our patients had routine psychiatric or cognitive assessment, meaning patients with subtle NP manifestations such as cognitive dysfunction might have been missed. Second, the treatment strategy was not standardised, but instead was individualised according to patients’ disease activity and manifestations which might confound treatment outcomes. Lastly, due to the retrospective nature of our study, there may be reporting, selection, and referral bias. Strengths of our study included low rates of follow-up loss and missing data.

## Conclusions

NPSLE occurs in about 12% of children with LN and has heterogeneous presentations. Severe kidney manifestation and non-adherence are associated with NPSLE. LN patients developing NPSLE are at risk of mortality and poor kidney outcomes. Judicious and timely use of immunosuppression would be important to attain disease remission and improve patient outcomes. Future multi-national multi-centre collaborative studies are warranted to properly inform the predictive factors and outcomes of NPSLE in the setting of childhood-onset LN.

## Supplementary Information

Below is the link to the electronic supplementary material.

Graphical abstract (PPTX 185 KB)

ESM1 (DOCX 24.4 KB)

## Data Availability

The datasets generated during and/or analysed during the current study are available from the corresponding author on reasonable request.
