# Supplementary material for: Neuropsychiatric SLE in children with childhood-onset lupus nephritis: a 20-year retrospective cohort study
Source: Pediatr Nephrol. 2025 Aug 26;41(1):89–100. doi: 10.1007/s00467-025-06904-0 (PMC12685967; doi:10.1007/s00467-025-06904-0)
Supplement: Supplementary file 1 — (PPTX 185 KB) [file 467_2025_6904_MOESM1_ESM.pptx]

## Slide 1
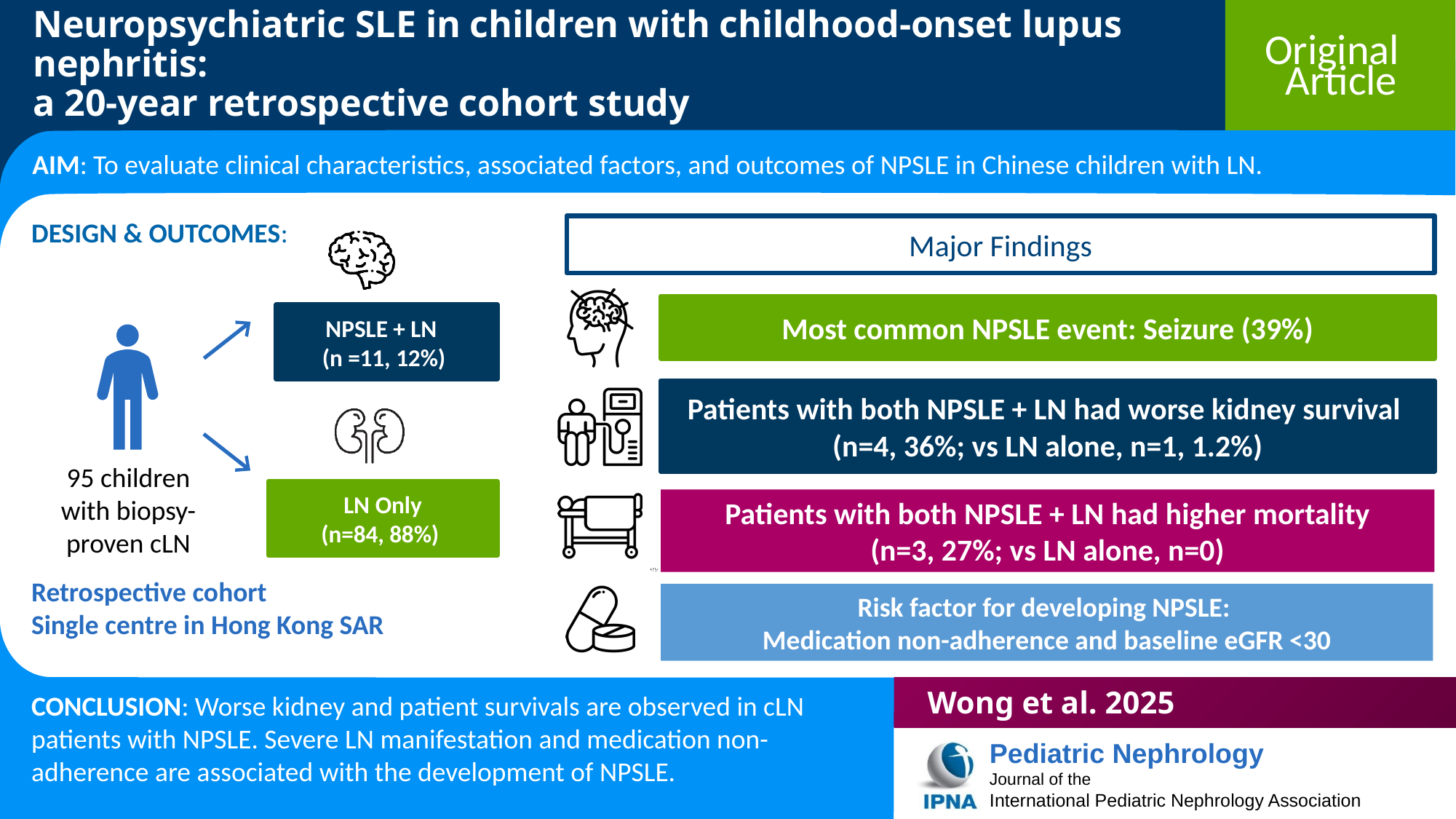

Neuropsychiatric SLE in children with childhood-onset lupus nephritis:
a 20-year retrospective cohort study
AIM: To evaluate clinical characteristics, associated factors, and outcomes of NPSLE in Chinese children with LN.
DESIGN & OUTCOMES:
Major Findings
Most common NPSLE event: Seizure (39%)
NPSLE + LN
(n =11, 12%)
Patients with both NPSLE + LN had worse kidney survival
(n=4, 36%; vs LN alone, n=1, 1.2%)
95 children with biopsy-proven cLN
LN Only
(n=84, 88%)
Patients with both NPSLE + LN had higher mortality
(n=3, 27%; vs LN alone, n=0)
Retrospective cohort
Single centre in Hong Kong SAR
Risk factor for developing NPSLE:
Medication non-adherence and baseline eGFR <30
Wong et al. 2025
CONCLUSION: Worse kidney and patient survivals are observed in cLN patients with NPSLE. Severe LN manifestation and medication non-adherence are associated with the development of NPSLE.
