# Supplementary material for: Neuropsychiatric SLE in children with childhood-onset lupus nephritis: a 20-year retrospective cohort study
Source: Pediatr Nephrol. 2025 Aug 26;41(1):89–100. doi: 10.1007/s00467-025-06904-0 (PMC12685967; doi:10.1007/s00467-025-06904-0)
Supplement: Supplementary file 2 — (DOCX 24.4 KB) [file 467_2025_6904_MOESM2_ESM.docx]

**Online resource**

**Neuropsychiatric SLE in children with childhood-onset lupus nephritis: A 20-year retrospective cohort study.

Matthew Lok-hei Wong FHKAM(Paed)^1^, Ka-Man Yip^3^, Alison Lap-tak Ma FRCPCH^2,3^, Eugene Yu-hin Chan FHKAM (Paed) FRCPCH^2^**

^1^Department of Paediatric and Adolescent Medicine, Princess Margaret Hospital, Hong Kong SAR

^2^Paediatric Nephrology Centre, Hong Kong Children’s Hospital, Hong Kong SAR
^3^Department of Paediatrics and Adolescent Medicine, The University of Hong Kong, Hong Kong SAR

Co-Corresponding authors:
Eugene Yu-hin Chan FRCPCH (UK), Email:eugene.chan@cuhk.edu.hk
Paediatric Nephrology Centre, Hong Kong Children’s Hospital, Hong Kong

**Supplementary Table S1: Baseline presentations and cumulative symptoms of 95 LN children with and without neuropsychiatric manifestation**

|  | Total (N=95) | With NPSLE (N=11) | Without NPSLE (N=84) | P value |
| --- | --- | --- | --- | --- |
| *Baseline presentations at diagnosis of cSLE* |  | | | |
| Renal | 80 (84%) | 8 (73%) | 72 (86%) | NS |
| Serositis | 12 (13%) | 2 (18%) | 10 (12%) | NS |
| Constitutional symptoms | 35 (37%) | 2 (18%) | 33 (39%) | NS |
| GI disease | 5 (5.3%) | 1 (9.1%) | 4 (4.8%) | NS |
| Haemolytic anemia | 66 (69%) | 5 (45%) | 61 (73%) | NS |
| Leukopenia | 42 (44%) | 3 (27%) | 39 (46%) | NS |
| Thrombocytopenia | 48 (51%) | 8 (73%) | 40 (48%) | NS |
| Mucocutaneous | 54 (57%) | 4 (36%) | 50 (60%) | NS |
| Arthritis/Arthralgia | 35 (37%) | 2 (18%) | 33 (39%) | NS |
| Ophthalmic | 1 (1.1%) | 0 | 1 (1.2%) | NS |
| APS | 2 (2.1%) | 0 | 2 (2.4%) | NS |
| Hypertension | 33 (35%) | 3 (27%) | 30 (36%) | NS |
| *Cumulative symptoms* |  | | | |
| Renal | 95 (100%) | 11 (100%) | 84 (100%) | NS |
| Serositis | 18 (19%) | 4 (36%) | 14 (17%) | NS |
| Constitutional symptoms | 35 (37%) | 2 (18%) | 33 (39%) | NS |
| GI disease | 12 (13%) | 1 (9%) | 11 (13%) | NS |
| Haemolytic anemia | 68 (72%) | 6 (55%) | 62 (74%) | NS |
| Leukopenia | 44 (46%) | 3 (27%) | 41 (49%) | NS |
| Thrombocytopenia | 50 (53%) | 8 (73%) | 42 (50%) | NS |
| Mucocutaneous | 59 (62%) | 4 (36%) | 55 (66%) | NS |
| Arthritis/arthralgia | 37 (39%) | 3 (27%) | 34 (40%) | NS |
| Ophthalmic | 5 (5.3%) | 1 (9.1%) | 4 (4.8%) | NS |
| APS | 4 (4.2%) | 2 (18%) | 2 (2.4%) | NS |
| Hypertension | 44 (46%) | 6 (55%) | 38 (45%) | NS |
| Value expressed as count (%)  Abbreviation: NPSLE: Neuropsychiatric systemic lupus erythematosus; cSLE: childhood onset systemic lupus erythematosus; GI: gastrointestinal; AKI: acute kidney injury  APS: anti-phospholipid syndrome  NS = Not statistically significant, p value > 0.05 | | | | |

**Supplementary Table S2:**

**Neurological investigations in 11 patients with neuropsychiatric manifestations**

| Investigations | Abnormalities* | Abnormal findings (N)^ |
| --- | --- | --- |
| CT brain | 7/10 (70%) | Infarction (3), ICH (1), vasculitic changes (2), cerebral atrophy (1) |
| MRI brain | 10/11 (91%) | Infarction (4), ICH (2), vasculitic changes (3), cerebral atrophy (1), inflammatory changes (1)transtentorial/cerebellar herniation (2)  dural sinus thrombosis (1), hydrocephalus # (1), midline shift #(1) |
| MRI spine | 1/1 (100%) | Atrophy changes of spine (1) |
| CSF abnormality | 3/5 (60%) | CSF protein elevation (3) |
| EEG abnormality | 4/4 (100%) | slow wave (4) |
| NCS abnormality | 0/1 |  |
| * Number of abnormal/ total number of investigations (%)  ^ One abnormal investigation may have more than one abnormal findings  # Complication of severe ICH  Abbreviation: CT: Computed tomography; MRI: Magnetic Resonance Imaging; CSF: Cerebrospinal fluid; EEG: Electroencephalography; NCS: Nerve conduction study; ICH: intracranial haemorrhage | | |

**Supplementary Table S3 : Comparison of laboratory parameters of lupus nephritis children with and without neuropsychiatric manifestations**

|  | With NPSLE (N=11) | Without NPSLE (N=84) | P value |
| --- | --- | --- | --- |
| *Laboratory parameters at diagnosis of LN* | | | |
| Urine protein creatinine ratio (mg/mg) | 3.4(1.7-8.1) | 2.4 (0.8-5.5) | NS |
| Serum creatinine (umol/L) | 63 (51-97) | 56 (44-81) | NS |
| eGFR <30 (ml/min/1.73m^2^) | **3 (27%)** | **5 (6%)** | ***0.048** |
| eGFR >=30 (ml/min/1.73m^2^) | 8 (73%) | 79 (94%) |  |
| anti-dsDNA (IU/ml) | 200 (58-250) | 200 (199-250) | NS |
| C3 (g/L) | 0.3 (0.24-0.54) | 0.39 (0.26-0.52) | NS |
| ESR (mm/hour) | 34 (18-49) | 40 (21-60) | NS |
| SLEDAI-2K score | 21 (17-31) | 16.5 (14-22) | NS |
| *Laboratory parameters during follow up* | | | |
| Highest urine protein creatinine ratio (mg/mg) | **6.6 (2.6-13.7)** | **3.3 (1.7-6.7)** | ***0.044** |
| Highest serum creatinine (umol/L) | 93 (76-151) | 83 (64-128) | NS |
| Highest anti-dsDNA (IU/ml) | 200 (200-250) | 200 (200-250) | NS |
| Lowest C3 (g/L) | 0.31 (0.23-0.42) | 0.37 (0.24-0.49) | NS |
| Lowest Platelets, x10^9^/L | 73 (48-135) | 140 (80-198) | NS |
| *Auto-antibodies profile* | | | |
| Anti-Ro seropositivity | 4 (36%) | 44 (52%) | NS |
| Anti-La seropositivity | 1 (9%) | 14 (17%) | NS |
| Anti-ribosomal P seropositivity | 3 (27%) | 28 (33%) | NS |
| Anti-smith seropositivity | 2 (18%) | 18 (21%) | NS |
| LA/anti-cardiolipin IgG/IgM seropositivity | 2 (18%) | 42 (50%) | NS |
| Value expressed as count (%), median (IQR)  *p <0.05: statistically significant;  Abbreviation: NPSLE: Neuropsychiatric systemic lupus erythematosus ; LN: lupus nephritis ; eGFR: estimated glomerular filtration rate ; LA: lupus anticoagulant ; ESR: Erythrocyte sedimentation rate; Anti dsDNA: anti double stranded DNA titre; NS: Not statistically significant, p value > 0.05 | | | |
